# Insights into Comparative Genomics, Codon Usage Bias, and Phylogenetic Relationship of Species from Biebersteiniaceae and Nitrariaceae Based on Complete Chloroplast Genomes

**DOI:** 10.3390/plants9111605

**Published:** 2020-11-18

**Authors:** Xiaofeng Chi, Faqi Zhang, Qi Dong, Shilong Chen

**Affiliations:** 1 Key Laboratory of Adaptation and Evolution of Plateau Biota, Northwest Institute of Plateau Biology, Chinese Academy of Sciences, Xining 810008, China; xfchi@nwipb.cas.cn (X.C.); fqzhang@nwipb.cas.cn (F.Z.); 2 Qinghai Provincial Key Laboratory of Crop Molecular Breeding, Northwest Institute of Plateau Biology, Chinese Academy of Sciences, Xining 810008, China

**Keywords:** Biebersteiniaceae, Nitrariaceae, chloroplast genome, codon usage bias, phylogenetic analysis

## Abstract

Biebersteiniaceae and Nitrariaceae, two small families, were classified in Sapindales recently. Taxonomic and phylogenetic relationships within Sapindales are still poorly resolved and controversial. In current study, we compared the chloroplast genomes of five species (*Biebersteinia heterostemon, Peganum harmala, Nitraria roborowskii, Nitraria sibirica,* and *Nitraria tangutorum*) from Biebersteiniaceae and Nitrariaceae. High similarity was detected in the gene order, content and orientation of the five chloroplast genomes; 13 highly variable regions were identified among the five species. An accelerated substitution rate was found in the protein-coding genes, especially *clpP*. The effective number of codons (EN_C_), parity rule 2 (PR2), and neutrality plots together revealed that the codon usage bias is affected by mutation and selection. The phylogenetic analysis strongly supported (Nitrariaceae (Biebersteiniaceae + The Rest)) relationships in Sapindales. Our findings can provide useful information for analyzing phylogeny and molecular evolution within Biebersteiniaceae and Nitrariaceae.

## 1. Introduction

Biebersteiniaceae and Nitrariaceae are two small families. Biebersteiniaceae consists of only one genus *Biebersteinia*, which comprises 4–5 species, and is mainly distributed in the eastern Mediterranean, Central, and Western Asia, northwest of China, and the Himalayas [1,2]. Some species of *Biebersteinia* was used as traditional folk medicines in Iran and China with the activities of anti-inflammatory, analgesic, antibacterial, antioxidant, antihypertensive, and hypoglycemic effects [3]. Nitrariaceae consists of four genera, namely *Nitraria*, *Tetradiclis, Malacocarpus,* and *Peganum*, which comprise about 20 species, and are native to arid and semi-arid regions of Mexico, North Africa, Europe, Australia, and Asia [1,4]. The plants of *Nitraria* genus are highly adaptable to high salinity, arid, or semi-arid environments. They can effectively alleviate the salinization degree of soil, improve the utilization rate of saline-alkali land and prevent soil desertification [5].

The placement of the five genera (*Biebersteinia, Nitraria*, *Tetradiclis, Malacocarpus,* and *Peganum*) have long been controversial [6]. *Biebersteinia* was traditionally placed in Geraniaceae or near Geraniaceae as a distinct family [7]. However, molecular evidence from recent studies supported a strong position for Biebersteiniaceae within Sapindales [7,8]. In earlier classifications, the position of *Nitraria*, *Tetradiclis, Malacocarpus,* and *Peganum* varied tremendously, and most researchers followed Engler’s system, where these genera were in Zygophyllaceae [4]. However, molecular phylogenetic studies have shown that the four genera were more closely related to Sapindales than Zygophyllaceae [8,9,10]. The relationships of Biebersteiniaceae and Nitrariaceae with regard to the other families of Sapindales is not resolved with confidence due to the sampling size, as well as the DNA regions used in the phylogenetic analysis [8,10,11,12,13,14].

Molecular phylogeny applies specific gene sequences to reconstruct the evolutionary history of organisms and provides resolutions for phylogeny of some taxonomically difficult groups [15]. Chloroplast DNA sequences were widely used in plant phylogenetic research, due to their abundant variation sites, moderate nucleotide substitution rates and no interference of paralogy [16]. However, with the accumulation of molecular evidence, the results of molecular phylogenetic studies on the same group based on different molecular fragments sometimes are conflict [17]. As a result, phylogenomics using chloroplast genomes data have developed rapidly in recent years and have been successfully applied to resolve many enigmatic relationships within angiosperms [13,18,19].

Chloroplasts are important plant organelles that play a vital role in photosynthesis, biosynthesis, and carbon sequestration [20]. Chloroplasts have a genetic system independent of the nuclear genome. Since the first chloroplast genome from *Nicotiana tabacum* [21], the structure and function have been increasingly understood. The chloroplast genome, which ranges from 100 kb to 200 kb, has a typical four-part structure, including the large-single copy (LSC) region, the small-single copy (SSC) region and two inverted repeat regions (IR) [22]. Advances in next-generation sequencing technologies have led to the complete sequencing of many chloroplast genomes; more than two thousand have been deposited in the National Center for Biotechnology Information (NCBI).

Codon usage bias refers to the differences in the frequency of synonymous codons that code for the same amino acid. The phenomenon is common in organisms from prokaryotes to unicellular and multicellular eukaryotes [23]. However, different genomes have their characteristic synonymous codon usage patterns, making it challenging to explain the bias [24]. Generally, codon usage bias reflects the origin, evolution and mutation patterns of the species or genes and has substantial effects on gene function and protein expression [25]. Previous studies focused on the codon usage bias in nuclear genomes [26,27]. As an evolutionary paradox with relatively conserved genes, chloroplast genomes’ genetic code differs from the standard genetic code [28]. Therefore, analysis of the codon usage bias in the chloroplast genomes will help understand the underlying molecular mechanisms of codon bias selection and the evolution and environmental adaptation of related species.

Therefore, in this study, we systematically present chloroplast genomes of five species from Biebersteiniaceae and Nitrariaceae. Our aims of this study were (1) to compare the chloroplast genomes and identify the variable regions; (2) to explore the codon usage patterns; and (3) to elucidate the phylogenetic relationship of Biebersteiniaceae and Nitrariaceae in Sapindales. The obtained results will provide a deeper understanding and classification of these two families in Sapindales.

## 2. Results

### 2.1. General Features of the Chloroplast Genomes

The length of the chloroplast genomes of the five species ranges from 158,795 bp (*B. heterostemon*) to 160,038 bp (*P. harmala*). The genomes have a typical structure with an LSC region (86,887–88,278 bp), an SSC region (18,309–18,852 bp) and two IR regions (26,469–26,779 bp; Table 1). The overall guanine and cytosine (GC) content of the genomes ranges from 37.15% to 37.90%. The IR regions have the highest GC content (42.67–42.79%), followed by the LSC regions (35.19–36.21%) and the SSC regions (31.42–32.0%). The *B. heterostemon* chloroplast genome contains 135 predicted genes, including 88 protein-coding genes, 39 tRNA genes, and 8 rRNA genes. All three *Nitraria* species’ genomes harbor 128 genes, including 81 protein-coding genes, 39 tRNA genes, and 8 rRNA genes. Meanwhile, the *P. harmala* genome comprises 132 annotated genes, including 87 protein-coding genes, 37 tRNA genes and 8 rRNA genes.

### 2.2. Genome Comparison

The analysis revealed high sequence similarity among the five species, suggesting they are conserved in size and structure. The *rpl22* gene was positioned at the LSC/IRb junction, with 44–75 bp in the IRb region (Figure S1). The *ndhF* gene was located at the IRb/SSC junction, with 0–18 bp in the IRb region. The SSC/IRa junction was crossed by *ycf1*, with 1298–1441 bp in the IRa region in all the five chloroplast genomes. The *rps19*-*trnH* genes were located at the IRa/LSC junction, with 17–62 bp separating the spacer from the end of the IRa region. Overall, IR region contraction/expansion events were detected across the five chloroplast genomes.

**Figure 1:**
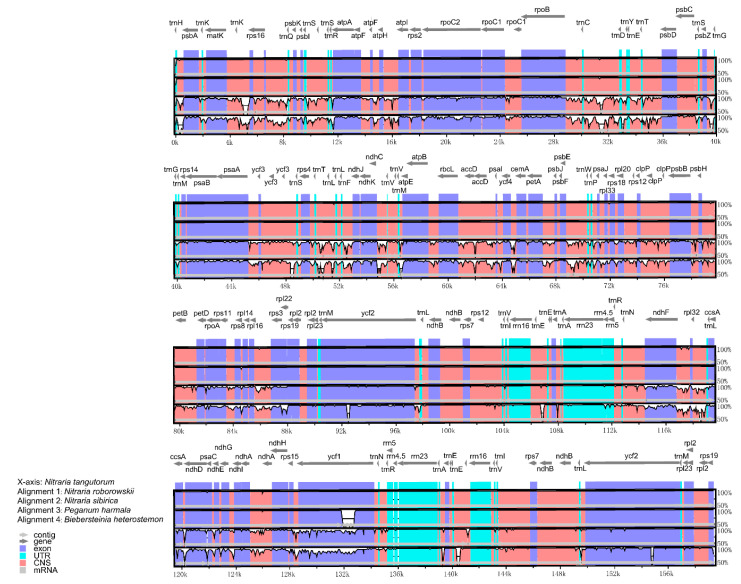
Comparison of five chloroplast genomes using the mVISTA alignment program with *N. roborowskii* as a reference. The *x*-axis represents the coordinates in the chloroplast genome. The *y*-axis indicates the average percent identity of sequence similarity in the aligned regions, ranging between 50% and 100%. Genome regions are color coded as protein coding, rRNA coding, tRNA coding, or conserved noncoding sequences (CNS).

To elucidate the genome divergence, we performed multiple sequence alignment of the five chloroplast genomes using the mVISTA program with *N. tangutorum* as the reference (Figure 1). The comparison demonstrated highly conserved coding regions compared with the non-coding regions among the five species. The IR regions were less divergent than the LSC and SSC regions. The highest divergence was found among the intergenic spacers, including *trnH*-*psbA*, *matK*-*rps16*, *rps16*-*psbK*, *psbI*-*atpA*, *rpoB*-*psbD*, *petN*-*psbM*, *psaA*-*rps4*, *rps4*-*ndhJ*, *ndhC*-*atpE*, *ycf4*-*cemA*, and *psbE*-*psaJ* in LSC and *ndhF*-*ccsA* and *ndhG*-*ndhI* in SSC. In addition, a greater sequence divergence was found in the coding regions of *matK*, *rpoA*, *rps19*, *ndhF*, *ccsA*, *ndhD*, and *ycf1*.

Furthermore, the nucleotide variability (Pi) was calculated to determine the sequence divergence levels among the five chloroplast genomes. Analysis revealed that the IR regions are more conserved than the LSC and SSC regions (Figure S2). We detected seven highly variable regions (Pi > 0.10), namely *trnH*-*psbA*, *matK*-*rps16*, *psbK*-*psbI*, *trnE*-*trnT*, *trnF*-*ndhJ*, *ndhD*-*ndhG*, and *rrn23*-*trnA*, which can be used as potential markers for further genetic studies.

### 2.3. Variation in Nucleotide Substitution Rates

**Figure 2:**
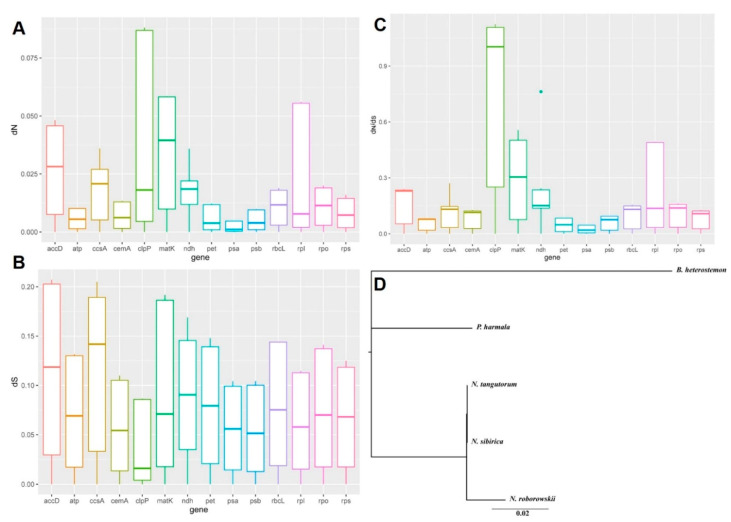
Nonsynonymous (*d_N_*) and synonymous (*d_S_*) substitution rates in the 74 protein-coding genes among the five species. (**A**): Nonsynonymous (*d_N_*) substitution rates in the 74 protein-coding genes; (**B**): Synonymous (*d_S_*) substitution rates in the 74 protein-coding genes; (**C**): *d_N_*/*d_S_* ratio of the 74 protein-coding genes; (**D**): Phylograms of the five species’ substitution rates based on 74 protein-coding genes.

Synonymous and nonsynonymous nucleotide substitution patterns are important markers used in the study of gene evolution. In most genes, except rapidly evolving genes, the frequency of nonsynonymous nucleotide substitution (*d_N_*) is lower than that of synonymous substitution (*d_S_*) due to purifying selection. Generally, d_N_/d_S_ < 1 (especially less than 0.5) indicates purifying selection; *d_N_*/*d_S_* > 1 indicates possible forward selection; and *d_N_*/*d_S_* value approaching 1 indicates neutral evolution [29]. The average *d_N_*/*d_S_* value of the five species’ 74 protein-coding genes was 0.2566 (Figure 2). The genes *atp*, *pet*, and *psb*, with an average *d_N_*/*d_S_* value between 0 and 0.1. The model averaging method in *d_N_*/*d_S_* calculator showed an average *d_N_*/*d_S_* > 1 for *clpP*.

### 2.4. Codon Usage Bias

**Figure 3:**
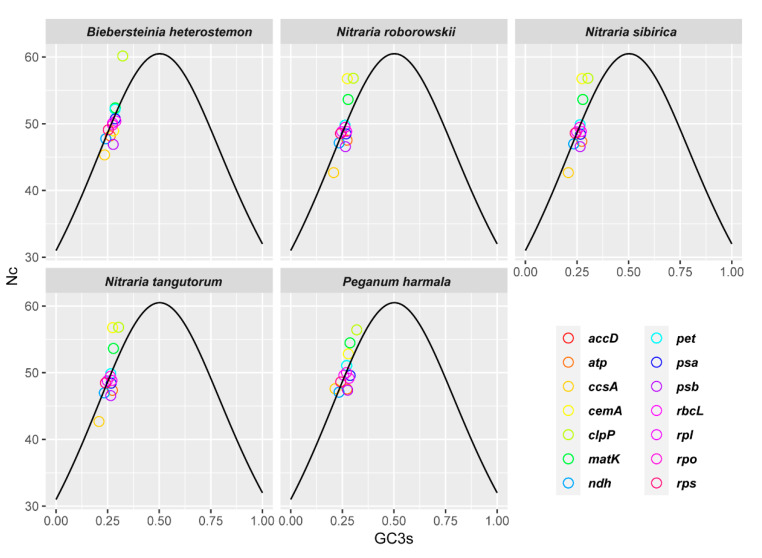
Effective number of codons (EN_C_) analysis of each coding gene against GC_3S_.

The total GC content (GC, 0.386–0.391), GC content at first codon position (GC_1_, 0.475–0.477), GC content at second codon position (GC_2_, 0.395–0.397), and GC content at third codon position (GC_3_, 0.287–0.300) less than 0.5 (Table 2) suggest that the five chloroplast genomes tend to use A/T bases and A/T-ending codons. The codon adaptation index (CAI) values between 0.167 and 0.170 indicate a slight bias in codon usage in the five species, especially *B. heterostemon*. The values of effective number of codons (EN_C_) ranged from 48.71 to 49.74. *B. heterostemon* had higher EN_C_ values than *P. harmala* and the three *Nitraria* species. Among the *Nitraria* species, *N. roborowskii* had higher EN_C_ values than *N. sibirica* and *N. tangutorum*. Further, to identify the forces that determine the five genomes’ overall codon usage, the EN_C_-GC_3S_ plot was drawn (Figure 3). Most genes lay close to Wright’s curve [30]. A few genes, including ccsA, with lower EN_C_ values, lay below the curve. 

**Figure 4:**
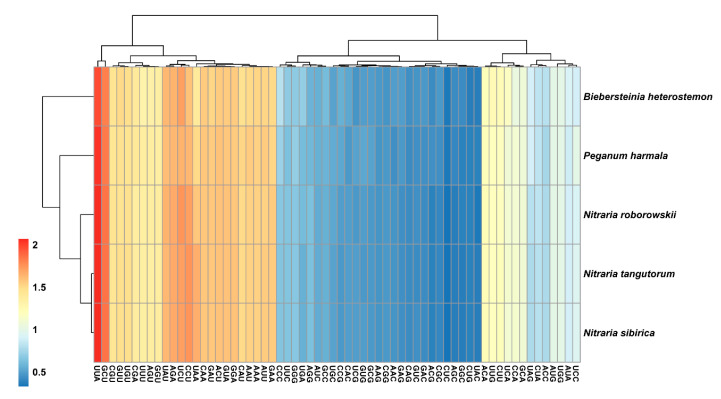
Heat map of relative synonymous codon usage (RSCU) values among the five species.

Slight differences were found in the relative synonymous codon usage (RSCU) among the five species (Figure 4). RSCU > 1 was found for 30 identical codons, of which 29 were A/T-ending codons (except for UUG) and RSCU < 1 for 32 identical codons, of which 29 were C/G-ending codons (except AUA, CUA and UGA). The highest RSCU value was recorded for UUA and the lowest for CUC, both encode leucine. To conclude, UUA was positively biased, while CUC was negatively biased.

**Figure 5:**
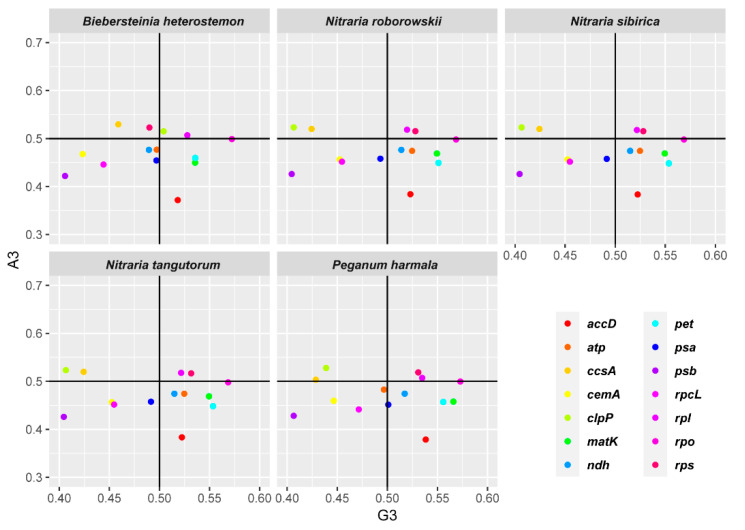
The parity rule 2 (PR2) bias plots (A_3_/(A_3_ + T_3_) against G_3_/(G_3_+ C_3_)).

A parity rule 2 (PR2) plot was generated based on AT-bias (A_3_*/*(A_3_ + T_3_)) and GC-bias (G_3_*/*(G_3_ + C_3_)) (Figure 5). The AT-biases of *B. heterostemon, P. harmala, N. roborowskii, N. sibirica,* and *N. tangutorum* were 0.475, 0.475, 0.476, 0.476, and 0.475, respectively, while the GC-biases were 0.501, 0.513, 0.511, 0.511, and 0.511, respectively. Thus, we detected T/G bias at the third codon position of chloroplast genes in the five species. However, different genes showed different preferences. The analysis revealed T/G bias in *accD*, *matK*, *ndh*, *pet*, and *rpo*; A/C bias in *ccsA*; T/C bias in *cemA*, *psa*, *psb*, and *rbcL*; and A/G bias in *rpl*. *B. heterostemon* showed different codon usage bias at the third codon position in *atp*, *clpP*, and *rps* (*p* < 0.05). The *atp* genes of *B. heterostemon* and *P. harmala* were T/C-biased, whereas those in *N. roborowskii, N. sibirica,* and *N. tangutorum* were T/G-biased. The *clpP* gene in *B. heterostemon* was A/G biased, while the genes in *P. harmala, N. roborowskii, N. sibirica,* and *N. tangutorum* were A/C biased. The *rps* in *B. heterostemon* was A/C biased, while the genes in *P. harmala, N. roborowskii, N. sibirica* and *N. tangutorum* were A/G bias.

**Figure 6:**
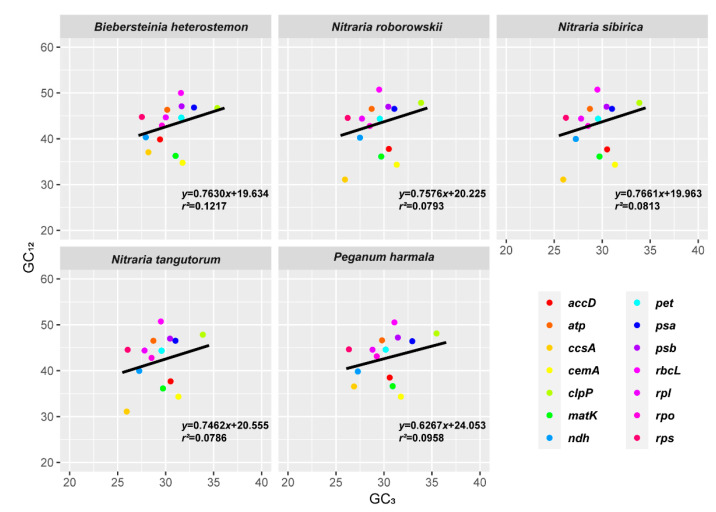
Neutrality plot (GC_12_ against GC_3_).

The difference in GC_3_ was more evident than that in GC_1_ and GC_2_ in the five genomes, reflecting the neutral mutation bias leading to different codon choice. The neutrality plots (GC_12_–GC_3_) were used to analyze the correlation between the three codon positions (Figure 6). The regression slopes of the five species were 0.7630 (*B. heterostemon),* 0.7576 (*N. roborowskii*), 0.7661 (*N. sibirica*), 0.7462 (*N. tangutorum*) and 0.6267 (*P. harmala*), with a correlation coefficient of 0.1217, 0.0793, 0.0813, 0.0786, and 0.0958, respectively.

### 2.5. Phylogenetic Analysis

**Figure 7:**
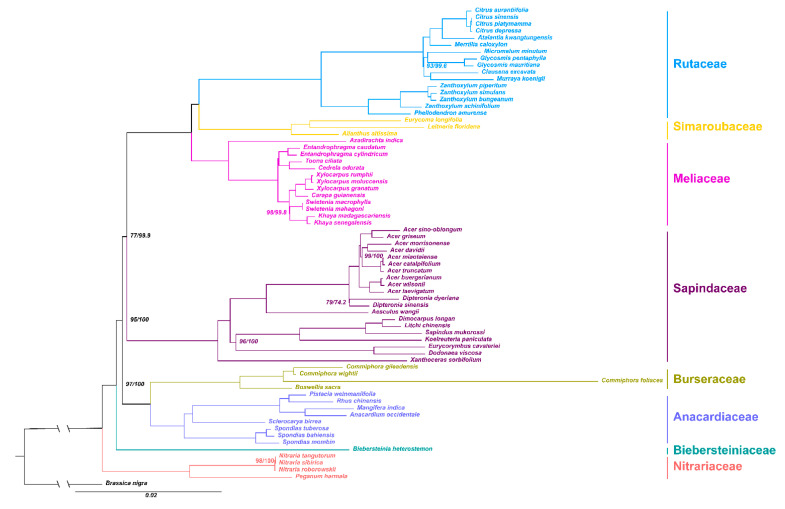
The phylogenetic relationships within Sapindales resolved by 67 protein-coding genes. Numbers associated with the branches are maximum likelihood (ML) bootstrap value (BS) and Bayesian inference (BI) posterior probabilities (PP). Nodes without numbers are supported by 100/100.

The maximum likelihood (ML) and Bayesian inference (BI) analysis of the whole plastome and 67 protein-coding genes dataset resulted in congruent topologies and only the nodes supporting was different (Figure 7 and Figure S3). We used the 67 protein-coding genes topology for the downstream analysis. Most clades were strongly supported by high bootstrap values (BS) and posterior probabilities (PP). Five well-supported clades were recovered within Sapindales in the phylogeny tree. Topology of (Nitrariaceae (Biebersteiniaceae + The Rest)) was supported in confidence. Biebersteiniaceae and Nitrariaceae was at the basal in Sapindales with a high support (BS = 97 and PP = 100). Anacardiaceae and Burseraceae together formed a strongly supported clade (BS = 100 and PP = 100). Meanwhile, the position of Sapindaceae was moderately supported (BS = 77 and PP = 100). Meliaceae, Simaroubaceae, and Rutaceae formed a clade with the highest support (BS = 100 and PP = 100). A sister relationship was observed between Rutaceae and Simaroubaceae, which was strongly supported (BS = 100 and PP = 100).

## 3. Discussion

### 3.1. Comparison of the Chloroplast Genomes

Highly-variable regions in the chloroplast genome can be used for the phylogenetic analysis and species discrimination. Studies have shown that genetic polymorphism in the single-copy regions (SSC and LSC) are more variable than the IR regions [31,32,33]. Similarly, in this study, the IR regions are more conserved than the LSC and SSC regions with a lower Pi value. Several researchers have conducted taxonomic studies; however, the taxonomic position and the phylogenetic relationship among the species of Biebersteiniaceae and Nitrariaceae remain poorly resolved [8,10,13,14]. Based on mVISTA and sliding window analysis, we identified the divergent regions in the chloroplast genomes that could be used as useful molecular markers. The variable regions *trnH-psbA*, *matK-rps16*, *rps16-psbK*, *psbI-atpA*, *rpoB-psbD*, *petN-psbM*, *psaA-rps4*, *rps4-ndhJ*, *ndhC-atpE*, *ycf4-cemA*, *psbE-psaJ*, *ndhF-ccsA,* and *ndhG-ndhI* would be ideal molecular markers to reconstruct the phylogenetic relationship in the Biebersteiniaceae and Nitrariaceae.

### 3.2. Variation in Nucleotide Substitution Rates

In photosynthetic angiosperms, the gene order, content, and rate of sequence evolution of protein-coding genes of the chloroplast genomes are generally conserved [34]. However, our analysis revealed variations in the substitution rate among the five species. Several protein-coding genes, including *accD*, *clpP*, *matK,* and *rpl*, showed accelerated *d_N_*, consistent with the previous reports [35,36,37,38]. Growing evidence suggests that the acceleration in gene-specific substitution rates may be due to local hypermutation or mutagenic retroprocessing [38]. The most extreme case was *clpP*, which had significantly higher *d_N_* (*d_N_* = 0.0973, *d_N_*/*d_S_* = 1.0611) in the present study. In angiosperm cells, *clpP* gene, comprise two introns and encode the ATP-dependent Clp protease proteolytic subunit. High substitution rates of the *clpP* gene was found in several species and mainly accompanied gene structural changes, including lack of the first intron or both the introns [38]. The amino acid sequence alignment of *clpP* gene (Figure S4) showed that *B. heterostemon* contains three large insertions that interrupted the conserved domains, which may induced the gene structural changes and a high *d_N_*/*d_S_* value.

### 3.3. Codon Usage Bias

Codon usage bias has been correlated with different factors, including gene expression level, GC content, amino acid conservation, and transcriptional selection [24]. Our study reveals selection and mutation as the probable mechanisms for codon bias. Selection theory explains that the codon bias contributes to the efficiency and/or accuracy of protein expression, and therefore undergoes positive selection. Meanwhile, the mutational explanation posits that codon bias exists due to the non-randomness in mutational patterns [25,39]. Although the mechanism behind codon bias selection remains controversial, a strong correlation is identified between the GC content and codon usage patterns in this study [24].

Furthermore, we found slight differences in the optimal codon usage patterns between the Biebersteiniaceae and Nitrariaceae species. The GC content of the protein-coding region (38.6–39.1%) is consistent with the whole genome (37.1–37.9%). The EN_C_ value reflects the codon bias level; EN_C_ value of 20 indicates maximum codon bias; a value of 61 indicates unbiased codon usage. A gene with an EN_C_ value of 35 or less is thought to possess strong codon bias. In current study, the EN_C_ values of the protein-coding genes ranged from 39 to 61; the majority were greater than 45, indicating a weak codon usage bias. According to EN_C_-plot analysis, the gene should fall on the standard curve in the graph when the codon use pattern is affected only by GC mutation. Nearly half of the genes were positioned on or close to the standard curve. The actual EN_C_ value closer to the theoretical EN_C_ value indicates that the codon usage is greatly affected by GC content, mutations. Few genes, especially *ccsA* and *psb* gene, lay below the standard curve. This finding confirms that codon usage is affected by selection, as reported in the previous studies [40,41,42].

GC content reflects the overall trend in mutation, and changes in the third base of the codon do not cause changes in the coding amino acid. Therefore, mutations in the third base are subject to low selection pressure. GC_3_ is also used to analyze the codon usage pattern. PR2 analysis used to determine the correlation between A and T and G and C in the third position of the codon showed that T was used slightly more frequently than A, while C was used more frequently than G, which indicates that pyrimidine was used more frequently than purine. Most of the mutations in the third position of the codon are synonymous mutations. The base changes in the first and second positions of the codon, which result in changes in the amino acids, are nonsynonymous mutations. Furthermore, the correlation between GC_12_ and GC_3_ was weak (*R*^2^ = 0.0786~0.1217), and the mutation has different effects on the composition of the first, second, and third positions of the codon. The third position of the codon is affected by weak mutation and other factors, such as selection, which may play an important role.

### 3.4. Phylogenetic Analysis within Sapindales

The developments in next-generation sequencing technologies have led to the sequencing of many chloroplast genomes. In addition, phylogenetic studies have been intensified, and many disputed relationships among plant species have been resolved [17]. In the present study, we obtained a robust phylogeny of (Nitrariaceae (Biebersteiniaceae + The Rest)) in the Sapindales. Phylogenetic relationships among Biebersteiniaceae and Nitrariaceae and related genera of the order Sapindales have long been controversial. The two-gene tree generated by Muellner et al., (2007) showed Biebersteiniaceae as a monophyletic group, with a possible sister relationship with eight Sapindales families [8]. Chen et al., (2016) found the (Nitrariaceae (Biebersteiniaceae + The Rest)) relationship; however, with little support (BS = 27) [14]. Some analyses supported (Nitrariaceae + Biebersteiniaceae) clade as sister to the rest of the order, the support was also poor [10,13]. Our results revealed that Nitrariaceae was at the basal of Sapindales followed by Biebersteiniaceae with strong support (BS = 97 and PP = 100). Meanwhile, A strong support for (Bursereae + Anacardiaceae) and (Meliaceae (Simaroubaceae + Rutaceae)) clades was also found, and such topology was supported by Soltis et al., 2011, Muellner-Riehl et al., 2016 and Li et al., 2019 [10,11,13]. The current phylogenomic study sheds new light on the disentangling of complex evolutionary events within Sapindales. Furthermore, inferences based on large-scale phylogenetic frameworks within Sapindales should benefit from phylogenetic genomics based upon whole-plastome sequencing.

## 4. Materials and Methods

### 4.1. Plant Materials and DNA Sequencing

*B. heterostemon* was collected from Xining Botanical Garden (101°44′43.03′′ E, 36°37′16.18′′ N, Qinghai, China), *P. harmala* was collected from Gonghe (100°41′41.74′′ E, 36°06′56.61′′ N, Qinghai, China), *N. roborowskii* (97°37′48.20′′ E, 37°16′27.21′′ N), *N. sibirica* (98°10′41.10′′ E, 36°59′51.60′′ N), and *N. tangutorum* (98°27′39.90′′ E, 36°56′05.73′′ N) were all collected from Haixi, Qinghai, China. All the specimens were deposited in the Qinghai–Tibetan Plateau Museum of Biology (HNWP). Total genomic DNA was extracted from the fresh leaves of one representative plant via the modified cetyltrimethylammonium bromide (CTAB) method [43] and measured by NanoDrop spectrophotometer (Thermo Scientific, Carlsbad, CA, USA). All the DNA samples were fragmented randomly, and paired-end libraries were constructed according to the Illumina preparation manual (San Diego, CA, USA). Universal primer and index primer for Illumina library amplification were listed in Table S1. Sequencing was performed on an Illumina HiSeq2500 platform (San Diego, CA, USA). Clean data was obtained by trimming the raw reads via the Trimmomatic tool [44].

### 4.2. Chloroplast Genome Assembling and Annotation

The chloroplast genome was assembled by SPAdes v 3.14 (https://cab.spbu.ru/software/spades/) [45]. Annotation was performed using the online tool CPGAVAS2 (http://47.96.249.172:16019/analyzer/home) [46] coupled with the manual adjustment of start/stop codons and intron/exon borders after BLAST searches. ARAGORN v1.2.38 (http://bioinfo.thep.lu.se) [47] was used to detect the transfer RNAs (tRNAs).

### 4.3. Genome Comparison

The online program IRscope (https://irscope.shinyapps.io/irapp/) [48] was used to check the contraction and expansion at the borders of IR regions. The mVISTA program (http://genome.lbl.gov/vista/index.shtml) [49] was employed in Shuffle-LAGAN mode to determine the differences among the chloroplast genomes of the five species. The nucleotide variability (average pairwise divergence; Pi) among the five chloroplast genomes was calculated via a sliding window analysis using DnaSP v5.10 (http://www.ub.edu/dnasp/) [50] with the following settings: 400 bp window length and 200 bp step size.

### 4.4. Estimation of Nucleotide Substitution Rate

Nucleotide substitution rates were estimated for 74 protein-coding genes which were shared by all five species (Table S2). The 74 genes were concatenated into 14 data sets: fatty acid synthesis (*accD*), ATP synthase (*atp*), cytochrome c synthesis (*ccsA*), carbon metabolism (*cemA*), proteolysis (*c1pP*), RNA processing (*matK*), NADPH dehydrogenase (*ndh*), cytochrome b_6_f (*pet*), photosystem I (*psa*), photosystem II (*psb*), RuBisCO (*rbcL*), RNA polymerase (*rpo*), large (*rpl*), and small (*rps*) ribosomal subunits. The data sets were aligned using MAFFT v7.0 (https://mafft.cbrc.jp/alignment/server/) [51]. Nonsynonymous (*d_N_*) and synonymous (*d_S_*) rates were calculated in PAML v4.9 (http://abacus.gene.ucl.ac.uk/software/paml.html) [52] using the codeml option with *F*3 × 4 codon frequencies. Box plots were drawn using ggplot2 in R v.3.6.3 (https://www.r-project.org/) to display the *d_N_* and *d_S_* values.

### 4.5. Codon Usage Bias

The level of codon usage bias was determined by calculating EN_C_, GC_3S_, CAI, RSCU, A_3_, U_3_, C_3_, and G_3_ using CodonW (http://codonw.sourceforge.net/) for all the protein-coding genes in the 14 data sets mentioned in Section 4.3. The EN_C_ vs. GC_3S_ plots and a heatmap of RSCU were generated based on the data sets. The PR2 plots were drawn based on the AU-bias (A_3_/(A_3_ + U_3_)) and GC-bias (G_3_/(G_3_ + C_3_)). The GC_1_, GC_2_, and GC_3_ was calculated in EMBOSS explorer (http://www.bioinformatics.nl/emboss-explorer/). The neutrality plot was drawn based on GC_3_ and GC_12_ (the average of GC_1_ and GC_2_). The heatmap and the different plots were drawn using ggplot2 in R v.3.6.3 (https://www.r-project.org/).

### 4.6. Phylogenetic Analysis

Apart from the five chloroplast genomes, 65 chloroplast genomes obtained from Genebank (Table S3) were used for the phylogenetic analysis. Among the 65 species, 64 species were from Sapindales, and *Brassica nigra* (Brassicaceae) was used as the outgroup. Phylogenetic analysis was conducted on the PhyloSuite v1.2.2 platform (https://dongzhang0725.github.io/) [53]. Nucleotide sequences of the whole chloroplast genomes and 67 protein-coding genes shared by all the 70 taxa sampled (Table S1) was firstly aligned in MAFFT based on default parameters. ModelFinder v1.6.8 (http://www.iqtree.org/ModelFinder/) [54] was used to select the best-fit model using AIC criterion. Maximum likelihood (ML) analyses were performed on the IQ-TREE v1.6.8 (http://www.iqtree.org/) [55] with 1000 bootstraps. Bayesian inference (BI) analyses was performed on the MrBayes v3.2.6 (http://nbisweden.github.io/MrBayes/) [56] with the number of generations (ngen) of 10,000,000, sample frequency (samplefreq) of 1000, and burnin (burninfrac) of 0.25. The generated trees were visualized using the online tool iTOL (Interactive Tree of Life; http://itol.embl.de/).

## 5. Conclusions

In present study, we compared the complete chloroplast genomes of five species from Biebersteiniaceae and Nitrariaceae. High similarity of structure and gene order, content was found among the five species. Several mutation hotspot regions for the five genomes included *trnH*-*psbA*, *matK*-*rps16*, *rps16*-*psbK*, *psbI*-*atpA*, *rpoB*-*psbD*, *petN*-*psbM*, *psaA*-*rps4*, *rps4*-*ndhJ*, *ndhC*-*atpE*, *ycf4*-*cemA*, *psbE*-*psaJ*, *ndhF*-*ccsA,* and *ndhG*-*ndhI*. Accelerated substitution rate was found in several protein-coding genes, including *accD*, *clpP*, *matK*, *rpl* and *clpP*, especially in *clpP*. The relative synonymous codon usage was biased among the five species, and the probable mechanisms may be selection and mutation. The phylogenetic analysis confirmed the topology of (Nitrariaceae (Biebersteiniaceae + The Rest)) in the Sapindales with strong support, providing a better-resolved phylogenetic relationship for the studied species of Sapindales than previous studies. Our findings reported here shed light on the structural evolution of chloroplast genomes and phylogenetic relationships of Biebersteiniaceae and Nitrariaceae.

## Figures and Tables

**Table 1:** The basic chloroplast genome characteristics.

Characteristics	*B. heterostemon*	*N. roborowskii*	*N. sibirica*	*N. tangutorum*	*P. harmala*
**Total cpDNA size (bp)**	158,795	159,397	159,364	159,383	160,068
**Length of large single copy (LSC) region**	86,887	87,907	87,883	87,901	88,278
**Length of inverted repeat (IR) region**	26,779	26,589	26,586	26,586	26,469
**Length of small single copy (SSC) region**	18,350	18,312	18,309	18,310	18,852
**Total GC content (%)**	37.90	37.32	37.27	37.15	37.50
**LSC**	36.21	35.20	35.20	35.19	35.60
**IR**	42.68	42.68	42.67	42.68	42.79
**SSC**	32.00	31.42	31.45	31.45	31.39
**Total number of genes**	135	128	128	128	132
**Protein-coding genes**	88	81	81	81	87
**tRNA genes**	39	39	39	39	37
**rRNA genes**	8	8	8	8	8

**Table 2:** Codon usage of the five species. GC: the total GC content; GC_1_: the GC content at first codon position; GC_2_: the GC content at second codon position; GC_3_: the GC content at third codon position; CAI: the codon adaptation index; T_3S_: the thymine content at synonymous third codon position; C_3S_: the cytosine content at synonymous third codon position; A_3S_: the adenine content at synonymous third codon position; G_3S_: the guanine content at synonymous third codon position; GC_3S_: the GC content at synonymous third codon position; EN_C_: the effective number of codons.

Species	GC	GC_1_	GC_2_	GC_3_	CAI	T_3S_	C_3S_	A_3S_	G_3S_	GC_3S_	EN_C_
*B. heterostemon*	0.391	0.477	0.397	0.300	0.170	0.464	0.172	0.420	0.173	0.269	49.74
*N. roborowskii*	0.389	0.477	0.396	0.295	0.168	0.468	0.165	0.423	0.174	0.264	49.38
*N. sibirica*	0.386	0.475	0.395	0.287	0.167	0.474	0.161	0.430	0.168	0.255	48.73
*N. tangutorum*	0.386	0.476	0.395	0.288	0.169	0.472	0.161	0.429	0.168	0.256	48.74
*P. harmala*	0.386	0.475	0.395	0.287	0.168	0.473	0.161	0.428	0.168	0.256	48.71

## Supplementary Materials

The following are available online at https://www.mdpi.com/2223-7747/9/11/1605/s1, Table S1. Primer used for Illumina library amplification, Table S2. Protein-coding genes used for nucleotide substitution rate, codon usage bias, and phylogenetic analysis, Table S3. Information of the species used for the phylogenetic analysis, Figure S1. Comparison of the borders of large single-copy (LSC), small single-copy (SSC), and inverted repeat (IR) regions among the chloroplast genomes of five species, Figure S2. Sliding window analysis of nucleotide variability (pairwise divergence) among the five species, Figure S3. The phylogenetic relationships within Sapindales resolved by complete chloroplast genome. Numbers associated with the branches are ML bootstrap value (BS) and BI posterior probabilities (PP). Nodes without numbers are supported by 100/1, Figure S4. Amino acid sequence alignment of *clpP* gene for the five species. Red box indicates the gene structural changes domains.
