# Supplementary material for: Insights into Comparative Genomics, Codon Usage Bias, and Phylogenetic Relationship of Species from Biebersteiniaceae and Nitrariaceae Based on Complete Chloroplast Genomes
Source: Plants (Basel). 2020 Nov 18;9(11):1605. doi: 10.3390/plants9111605 (PMC7699153; doi:10.3390/plants9111605)

Comparative Genomics, Codon Usage Bias and Phylogenetic Relationships of Species from Biebersteiniaceae and Nitrariaceae Based on Complete Chloroplast Genomes

Xiaofeng Chi[a,b], Qi Dong[a]*, Shilong Chen[a,b]*

[a] Key Laboratory of Adaptation and Evolution of Plateau Biota, Northwest Institute of Plateau Biology, Chinese Academy of Sciences, Xining 810008, China

[b] Qinghai Provincial Key Laboratory of Crop Molecular Breeding, Xining 810008, China

*For correspondence: Qi Dong, qdong@nwipb.cas.cn ; Shilong Chen, slchen@nwipb.cas.cn

Supplement Materials

Table S1. Primer used for Illumina library amplification.

Primer	Sequence
Universal Primer	5′-AATGATACGGCGACCACCGAGATCTACACTCTTTCCCTACACGACGCTCTTCCGATCT -3′
Index Primer	5′-CAAGCAGAAGACGGCATACGAGATGTGACTGGAGTTCAGACGTGTGCTCTTCCGATC-3′

Table S2. Protein-coding genes used for nucleotide substitution rate, codon usage bias and phylogenetic analysis.

Category	Name
Rubisco	*rbcL*
Photosystem I	*psaA, psaB, psaC, psaI, psaJ*
Photosystem II	*psbA, psbB, psbC**, *psbD, psbE, psbF, psbH, psbI, psbJ, psbK, psbL, psbM, psbN, psbT, psbZ*
ATP synthase	*atpA, atpB, atpE, atpF, atpH, atpI*
Cytochrome b/f complex	*petA, petB, petD, petG, petL, petN*
Cytochrome c synthesis	*ccsA*
NADPH dehydrogenase	*ndhA**, *ndhB, ndhC, ndhD, ndhE, ndhF, ndhG, ndhH, ndhI, ndhJ, ndhK*
Transcription	*rpoA, rpoB,rpoC1, rpoC2*
Ribosomal proteins	*rps2**,*rps3,rps4, rps7, rps8, rps11**, *rps12, rps14, rps15, rps16, rps18, rps19**, *rpl2, rp114, rp116, rp120, rp122**,*rp123, rp132 rp133, rp136*
RNA processing	*matK*
Carbon metabolism	*cemA*
Fatty acid synthesis	*accD**
Proteolysis	*c1pP*

* genes that not used for phylogenetic analysis

Table S3. Information of the species used for the phylogenetic analysis

NO.	Species	Genebank Accession	Family
1	*Anacardium occidentale*	KY635877.1	Anacardiaceae
2	*Mangifera indica*	KY635882.1	Anacardiaceae
3	*Pistacia weinmaniifolia*	MF630953.1	Anacardiaceae
4	*Rhus chinensis*	KX447140.1	Anacardiaceae
5	*Sclerocarya birrea*	MK002721.1	Anacardiaceae
6	*Spondias bahiensis*	KU756561.1	Anacardiaceae
7	*Spondias mombin*	KY828469.1	Anacardiaceae
8	*Spondias tuberosa*	KU756562.1	Anacardiaceae
9	*Biebersteinia heterostemon*	MN818816	Biebersteiniaceae
10	*Brassica nigra*	KT878383.1	Brassicaceae
11	*Boswellia sacra*	KT934315.1	Burseraceae
12	*Commiphora foliacea*	MH041484.1	Burseraceae
13	*Commiphora gileadensis*	MH042752.1	Burseraceae
14	*Commiphora wightii*	MF957201.1	Burseraceae
15	*Azadirachta indica*	KF986530.1	Meliaceae
16	*Carapa guianensis*	MF401522.1	Meliaceae
17	*Cedrela odorata*	MG724915.1	Meliaceae
18	*Entandrophragma caudatum*	MK058683.1	Meliaceae
19	*Entandrophragma cylindricum*	KY923074.1	Meliaceae
20	*Khaya madagascariensis*	MK058684.1	Meliaceae
21	*Khaya senegalensis*	KX364458.1	Meliaceae
22	*Swietenia macrophylla*	MH348156.1	Meliaceae
23	*Swietenia mahagoni*	NC_040009.1	Meliaceae
24	*Toona ciliata*	MG813875.1	Meliaceae
25	*Xylocarpus granatum*	MH348155.1	Meliaceae
26	*Xylocarpus moluccensis*	MH330688.1	Meliaceae
27	*Xylocarpus rumphii*	MH330687.1	Meliaceae
28	*Nitraria roborowskii*	MK347421	Nitrariaceae
29	*Nitraria sibirica*	MK347422	Nitrariaceae
30	*Nitraria tangutorum*	MK347423	Nitrariaceae
31	*Peganum harmala*	MK347420	Nitrariaceae
32	*Atalantia kwangtungensis*	MH329190.1	Rutaceae
33	*Citrus aurantiifolia*	KJ865401.1	Rutaceae
34	*Citrus depressa*	LC147381.1	Rutaceae
35	*Citrus platymamma*	KR259987.1	Rutaceae
36	*Citrus sinensis*	DQ864733.1	Rutaceae
37	*Clausena excavata*	KU949003.1	Rutaceae
38	*Glycosmis mauritiana*	KU949004.1	Rutaceae
39	*Glycosmis pentaphylla*	KU949005.1	Rutaceae
40	*Merrillia caloxylon*	KU949006.1	Rutaceae
41	*Micromelum minutum*	KU949007.1	Rutaceae
42	*Murraya koenigii*	KU949002.1	Rutaceae

43	*Phellodendron amurense*	KY707335.1	Rutaceae
44	*Zanthoxylum bungeanum*	KX497031.1	Rutaceae
45	*Zanthoxylum piperitum*	KT153018.1	Rutaceae
46	*Zanthoxylum schinifolium*	KT321318.1	Rutaceae
47	*Zanthoxylum simulans*	MF716524.1	Rutaceae
48	*Acer buergerianum*	KY419137.1	Sapindaceae
49	*Acer catalpifolium*	MF179637.1	Sapindaceae
50	*Acer davidii*	MK193786.1	Sapindaceae
51	*Acer griseum*	KY511609.1	Sapindaceae
52	*Acer laevigatum*	MF521832.1	Sapindaceae
53	*Acer miaotaiense*	KX098452.1	Sapindaceae
54	*Acer morrisonense*	KT970611.1	Sapindaceae
55	*Acer sino-oblongum*	KY987160.1	Sapindaceae
56	*Acer truncatum*	MF996341.1	Sapindaceae
57	*Acer wilsonii*	MG012225.1	Sapindaceae
58	*Aesculus wangii*	MF583747.1	Sapindaceae
59	*Dimocarpus longan*	MG214255.1	Sapindaceae
60	*Dipteronia dyeriana*	KT985457.1	Sapindaceae
61	*Dipteronia sinensis*	KT878501.1	Sapindaceae
62	*Dodonaea viscosa*	MF155892.1	Sapindaceae
63	*Eurycorymbus cavaleriei*	MG813997.1	Sapindaceae
64	*Koelreuteria paniculata*	KY859413.1	Sapindaceae
65	*Litchi chinensis*	KY635881.1	Sapindaceae
66	*Sapindus mukorossi*	KM454982.1	Sapindaceae
67	*Xanthoceras sorbifolium*	KY779850.1	Sapindaceae
68	*Ailanthus altissima*	MG799542.1	Simaroubaceae
69	*Eurycoma longifolia*	MH751519.1	Simaroubaceae
70	*Leitneria floridana*	KT692940.1	Simaroubaceae

Figure S1. Comparison of the borders of large single-copy (LSC), small single-copy (SSC), and inverted repeat (IR) regions among the chloroplast genomes of five species. JLB：junctions of LSC and IRb; JSB: junctions of SSC and IRb; JSA: junctions of SSC and Ira; JLA: junctions of LSC and IRa.

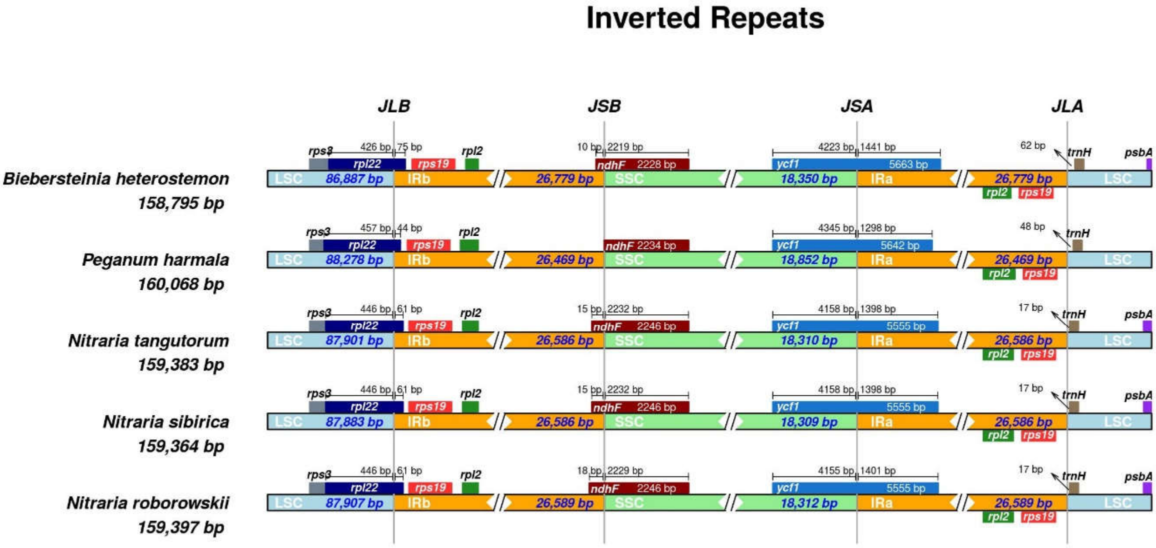

Figure S2. Sliding window analysis of nucleotide variability (pairwise divergence) among the five species. The number represented highly variable regions (Pi > 0.10). 1: trnH-psbA, 2: *matK-rps16*, 3: *psbK-psbI*, 4: *trnE-trnT*, 5: *trnF-ndhJ*, 6: *ndhD-ndhG*, and 7: *rrn23-trnA*.

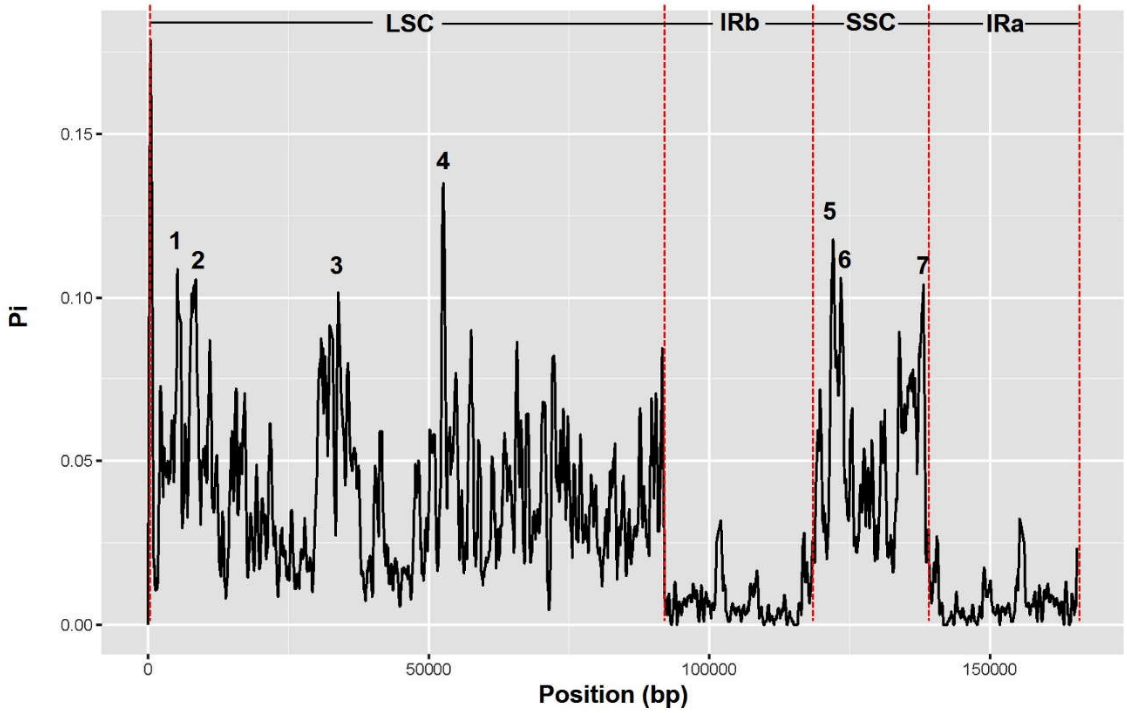

Figure S3. The phylogenetic relationships within Sapindales resolved by complete chloroplast genome. Numbers associated with the branches are ML bootstrap value (BS) and BI posterior probabilities (PP). Nodes without numbers are supported by 100/1.

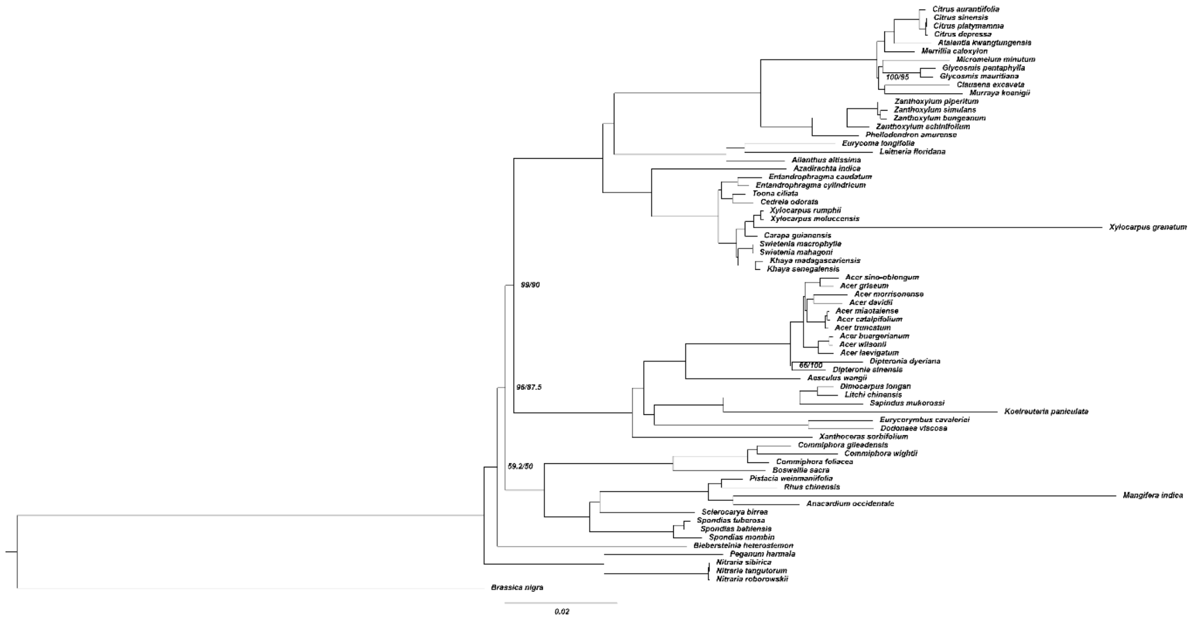

Figure S4. Amino acid sequence alignment of *clpP* gene for the five species. Red box indicates the gene structural changes domains.

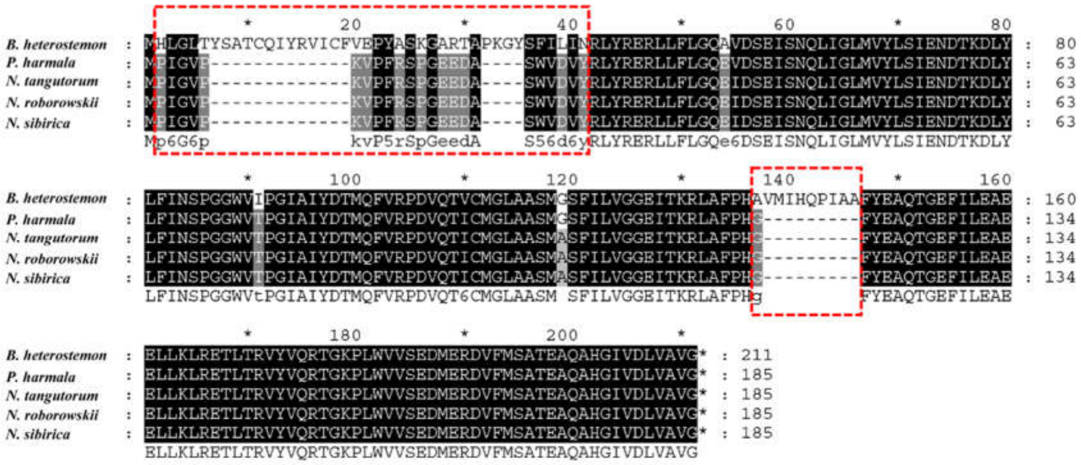

Supplement: Supplementary file 1 [file plants-09-01605-s001.pdf]
